# A microRNA Expression Profile as Non-Invasive Biomarker in a Large Arrhythmogenic Cardiomyopathy Cohort

**DOI:** 10.3390/ijms21041536

**Published:** 2020-02-24

**Authors:** Maria Bueno Marinas, Rudy Celeghin, Marco Cason, Riccardo Bariani, Anna Chiara Frigo, Joanna Jager, Petros Syrris, Perry M. Elliott, Barbara Bauce, Gaetano Thiene, Domenico Corrado, Cristina Basso, Kalliopi Pilichou

**Affiliations:** 1 Department of Cardiac-Thoracic-Vascular Sciences and Public Health, University of Padua, Cardiovascular Pathology, Cardiology and Biostatistics Units, 35121 Padua, Italy; maria.buenomarinas@unipd.it (M.B.M.); rudy.celeghin@gmail.com (R.C.); marco.cason@unipd.it (M.C.); bariani.riccardo@gmail.com (R.B.); annachiara.frigo@unipd.it (A.C.F.); barbara.bauce@unipd.it (B.B.); gaetano.thiene@unipd.it (G.T.); domenico.corrado@unipd.it (D.C.); cristina.basso@unipd.it (C.B.); 2 Centre for Heart Muscle Disease, Institute of Cardiovascular Science, University College London, London WC1E 6BT, UK; j.jager@ucl.ac.uk (J.J.); p.syrris@ucl.ac.uk (P.S.); perry.elliott@ucl.ac.uk (P.M.E.)

**Keywords:** microRNA 1, Arrhythmogenic Cardiomyopathy 2, biomarker 3

## Abstract

Arrhythmogenic Cardiomyopathy (AC) is a clinically and genetically heterogeneous myocardial disease. Half of AC patients harbour private desmosomal gene variants. Although microRNAs (miRNAs) have emerged as key regulator molecules in cardiovascular diseases and their involvement, correlated to phenotypic variability or to non-invasive biomarkers, has been advanced also in AC, no data are available in larger disease cohorts. Here, we propose the largest AC cohort unbiased by technical and biological factors. MiRNA profiling on nine right ventricular tissue, nine blood samples of AC patients, and four controls highlighted 10 differentially expressed miRNAs in common. Six of these were validated in a 90-AC patient cohort independent from genetic status: miR-122-5p, miR-133a-3p, miR-133b, miR-142-3p, miR-182-5p, and miR-183-5p. This six-miRNA set showed high discriminatory diagnostic power in AC patients when compared to controls (AUC-0.995), non-affected family members of AC probands carrying a desmosomal pathogenic variant (AUC-0.825), and other cardiomyopathy groups (Hypertrophic Cardiomyopathy: AUC-0.804, Dilated Cardiomyopathy: AUC-0.917, Brugada Syndrome: AUC-0.981, myocarditis: AUC-0.978). AC-related signalling pathways were targeted by this set of miRNAs. A unique set of six-miRNAs was found both in heart-tissue and blood samples of AC probands, supporting its involvement in disease pathogenesis and its possible role as a non-invasive AC diagnostic biomarker.

## 1. Introduction

Arrhythmogenic cardiomyopathy (AC, OMIM 107970, ORPHA247, ICD-10 I42.8) is a rare inherited heart muscle disease that is morphologically characterized by progressive myocardial dystrophy with fibro-fatty replacement [1,2,3] and clinically by life-threatening ventricular arrhythmias, leading to sudden cardiac death, particularly in the young and athletes [4,5].

AC is genetically determined by pathogenic variants in genes encoding proteins of the desmosomal complex, mainly inherited as autosomal dominant traits. Approximately half of the AC probands carry one or more point-mutations and copy-number variations in plakophilin-2 (*PKP2*), desmoplakin (*DSP*), desmoglein-2 (*DSG2*), desmocollin-2 (*DSC2*), and plakoglobin (*JUP*) [6]. AC exhibits an incomplete and age-related penetrance with variable expressivity making early clinical diagnosis challenging. The epigenetic and environmental factors that intervene as disease modifiers need to be better explored. 

MicroRNAs (miRNA) are a class of noncoding RNA, which undergo a maturation process that starts from the first transcribed molecule into the nucleus (pri-miRNA) to the effective and mature short miRNA of about 22 nt in the cytoplasm [7]. Their key function is gene-expression regulation by binding a specific messenger RNA (mRNA) that is based on their base-complementarity and degrading it or blocking its translation, leading to the inhibition of mRNA translation into protein [8]. 

**Figure 1:**
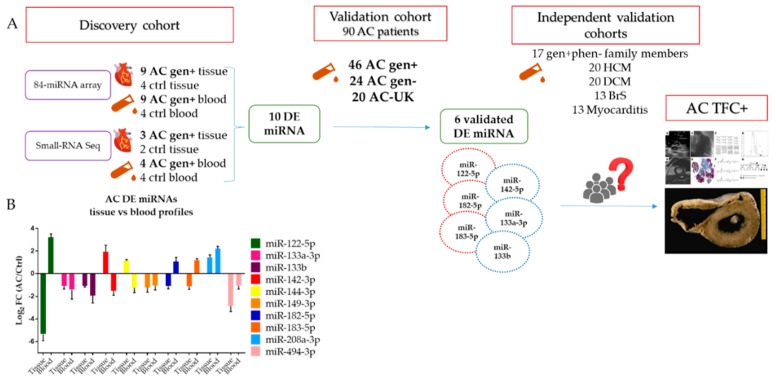
**(A**). Flow-chart of the entire cohort analysed, specifying the experimental steps performed; gen+: AC index cases carriers of a rare variant in a desmosomal gene; gen−: AC index cases genotype negative; AC-UK: AC index cases from the University College of London-UCL; gen+phen−: non-affected family members of AC probands carrying a desmosomal pathogenic variant; HCM: hypertrophic cardiomyopathy; DCM: dilated cardiomyopathy; BrS: Brugada syndrome; TFC: Task Force Criteria (**B**). Common tissue and blood profile in the discovery cohort: Log_2_FC mean ± SEM values of the 10 differentially expressed miRNAs.

Several biological roles were recognized in these noncoding RNAs, both in normal and pathological conditions, since miRNAs first discovery in 1993 in the nematode worm *Caenorhabditis elegans* (*C. elegans*) [9]. Aberrant miRNA expression has been investigated, not only in oncologic conditions, in which they regulate tumour suppressor genes and oncogenes [10,11], but also in different cardiovascular diseases [12,13,14]. As regards primary cardiomyopathies, the role of circulating miRNAs has been investigated for Hypertrophic Cardiomyopathy (HCM) [15,16,17,18], Dilated Cardiomyopathy (DCM) [19,20,21], and AC [22,23,24,25], showing remarkable stability under extreme conditions and suggesting their implication not only in cell-cell communication (as selectively secreted molecules), but also as a response to tissue-damage (as passive released molecules) in the onset and development of the disease [12]. Thus, the potential of circulating miRNAs as disease biomarkers is promising, given their function and localization inside and outside the cells in microvesicles, exosomes, apoptotic bodies, or associated with RNA-binding proteins. This study (Figure 1) focuses on the identification of a specific miRNA expression profile in AC and miRNAs as potential diagnostic biomarkers. 

## 2. Results

### 2.1. 84 miRNA Cardiac-Related Array Screening on Myocardial Tissue of AC Patients

Cardiac-related miRNA array analysis on myocardial samples from nine heart transplanted (HTx) AC index cases (Table 1, ID = AC1–AC9) of the discovery cohort (Figure 1) as compared to four controls (*ctrl*) showed 28 differentially expressed (DE) miRNAs in AC patients (AC-tissue profile), 12 overexpressed and 16 underexpressed (Supplementary Material, Figure S1). The nine AC HTx patients were carriers of a rare variant in a desmosomal gene (*gen+*) and subsequently subdivided into three groups that were based on the mutated gene in order to determine the presence of gene-specific profile. Specifically, *PKP2*-carriers of pathogenic variants displayed 34 DE miRNAs (PKP2 profile), 17 overexpressed and 17 underexpressed; *DSP*-carriers exhibited 31 DE miRNAs (DSP profile), 14 overexpressed and 17 underexpressed; and, *DSG2*-carriers (DSG2 profile) showed 16 DE miRNAs, seven overexpressed and nine underexpressed (Supplementary Material, Figure S2). Of note, PKP2 profile and DSP profile shared 26 DE miRNAs: 13 overexpressed and 13 underexpressed. All of the DE miRNAs found in the gene-dependent profiles were also present in the gene-independent miRNAs analysis of HTx AC patients (Supplementary Material, Figure S1). 

### 2.2. Small RNA-Seq on Myocardial Tissue of AC Patients

The AC-tissue profile of miRNAs was then validated in three of the nine HTx AC *gen+* patients (ID = AC1–AC3) and 2 *ctrl*. Small RNA-seq detected 860 known mature miRNAs with a minimum of 10 reads in all of the samples. Ninety-eight DE miRNAs were identified in AC, of which 59 miRNAs were significantly overexpressed and 39 significantly underexpressed (Log_2_FC cut-off >1 and <−1, respectively, and adjusted *p* value < 0.05). Specifically, 21 of the 28 miRNAs identified by array screening also showed consistent directional expression by Next Generation Sequencing-NGS analysis (let-7b-5p, miR-122-5p, miR-10b-5p, miR-133a-3p, miR-133b, miR-142-3p, miR-144-3p, miR-149-5p, miR-182-5p, miR-183-5p, miR-208a-3p, miR-21-5p, miR-210-3p, miR-223-3p, miR-26b-5p, miR-29b-3p, miR-320a, miR-328-3p, miR-378a-3p, miR-423-3p, and miR-494-3p).

### 2.3. 84 miRNA Cardiac-Related Array Screening in the Blood of AC Patients

The 84 miRNA cardiac-related array screening was also performed in whole blood samples of nine AC *gen+* patients from the discovery cohort (Table 1, ID = AC1, AC2, AC10–AC16), of whom two (ID = AC1, AC2) also had tissue-profiling (84 miRNA cardiac-related array and Small RNA-seq), and 4 *ctrl*, in order to investigate the circulating miRNA profile of AC *gen+* patients. We detected 15 DE miRNAs compared to *ctrl* (AC-blood profile), six significantly overexpressed and nine significantly underexpressed (Log_2_FC cut-off >1 and <−1, respectively) (Supplementary Material, Figure S1). No gene-dependent miRNA expression profile was observed in circulation. 

### 2.4. Small RNA-Seq on Blood of AC Patients

The AC-blood profile of miRNAs was then validated in 4 of the 9 AC *gen+* patients (ID = AC1, AC2, AC10-11) and 4 additional *ctrl*. Small RNA-seq detected 212 DE miRNAs in AC, of which 152 miRNAs were significantly overexpressed and 60 significantly underexpressed (Log_2_FC cut-off >1 and <−1, respectively, and adjusted *p* value < 0.05). Specifically, 11 of the 15 miRNAs that were identified by array screening were DE also by NGS analysis with consistent directionality (let-7a-5p, miR-122-5p, miR-133a-3p, miR-142-3p, miR-144-3p, miR-107, miR-182-5p, miR-183-5p, miR-22-3p, miR-31-5p, and miR-494-3p). 

### 2.5. Shared miRNA between AC- Tissue and AC-Blood Profiles

The intersection of DE miRNAs in the AC- tissue and AC-blood profiles led to the identification of 10 DE miRNAs in common in AC *gen*+ patients: miR-122-5p, miR-133a-3p, miR-133b, miR-142-3p, miR-144-3p, miR-149-5p, miR-182-5p, miR-183-5p, miR-208a-3p, and miR-494-3p. Four miRNAs (miR-133a-3p, miR-133b, miR-149-5p, and miR-494-3p) were downregulated in both specimens. An inconsistent directional expression was observed in tissue and blood profiling, for miR-122-5p, miR-182-5p, and miR-183-5p, which were underexpressed in tissue samples and overexpressed in blood samples. On the contrary, miR-142-3p and miR-144-3p were overexpressed in the myocardium, but underexpressed in blood samples (Figure 1B; Supplementary Material, Table S2). 

### 2.6. Validation of DE miRNA in a Larger AC Gen+ Cohort

In a larger AC cohort comprising 46 *gen+* index cases fulfilling TFC (Supplementary Material, Table S1, #1-46) and 20 *ctrl,* 10 DE miRNAs shared between tissue and blood profiles were validated. In this setting, we investigated the expression level of three more miRNAs associated with AC in previous studies: miR-21-5p, miR-320a, and miR-184 [22,23,27], as were also found in the AC-tissue profile. Specifically, miR-21-5p was overexpressed in myocardial tissue AC samples (Log_2_FC 2.986 ± 0.83), but not in blood, similarly miR-320a was only underexpressed in myocardial tissue AC samples (Log_2_FC −1.210 ± 0.43) and miR-184 was only detected by NGS screening (Log_2_FC 0.52 ± 0.30).

Six of these 13 miRNAs analysed by qPCR were DE significantly with a *p* value of less than 0.05: miR-122-5p, miR-133a-3p, miR-133b, miR-142-3p, miR-182-5p, and miR-183-5p (Supplementary Table S3). 

### 2.7. DE miRNA in Independent Validation Cohorts

The expression level of the 6-miRNA panel was then measured in five independent groups: 24 AC index cases with no rare variants in AC-correlated genes (*gen−*) (Supplementary Material, Table S1, #47-70), 17 non-affected family members of AC probands carrying a desmosomal pathogenic variant (*gen+phen−*), 20 AC patients from the University College of London-UCL (AC-UK), 20 index cases affected by HCM, 20 by DCM, 13 by Brugada Syndrome (BrS), and 13 myocarditis patients.

**Figure 2:**
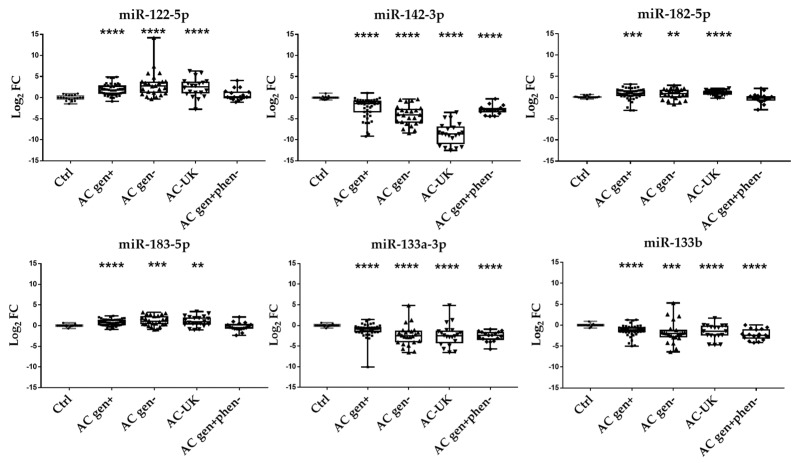
Box and whiskers plots and p values of the 6 significant DE miRNAs on the AC cohort: 46 AC gen+, 24 AC gen-, 20 AC-UK and 17 AC gen+phen− cohorts vs 20 ctrls (p value calculated by Mann-Whitney test, each group separately vs. ctrl: **** < 0.0001, *** < 0.001, ** < 0.01, * < 0.05).

The set of six miRNAs was observed DE with the same directionality as in AC *gen+* group only in the AC *gen−* and AC-UK groups, while the AC *gen+phen−* group only displayed only three of the six- miRNA panel dysregulated as in the AC group (miR-142-3p, miR-133a-3p, and miR-133b) (Figure 2). 

Interestingly, at least three miRNAs were significantly DE also in other cardiomyopathies: the HCM group showed miR-122-5p, miR-182-5p, and miR-183-5p overexpressed (*p* values < 0.05), the DCM group showed miR-122-5p overexpressed and miR-142-3p, miR-133a-3p, and miR-133b underexpressed (*p* values < 0.05), the BrS group showed miR- 142-3p, miR-182-5p, miR-133a-3p, and miR-133b underexpressed (*p* values < 0.05), and the myocarditis group showed miR-122-5p overexpressed and miR- 142-3p, miR-182-5p underexpressed (*p* values < 0.05) (Table 2).

### 2.8. 6-miRNA Panel as Potential Biomarker of AC

**Figure 3:**
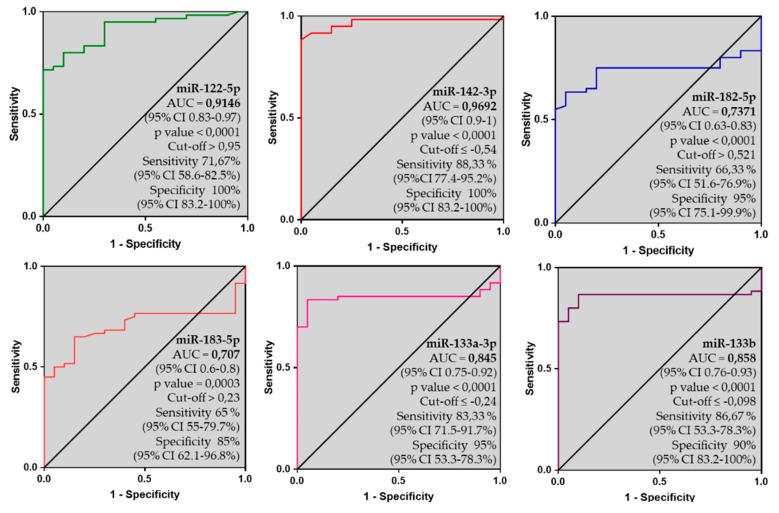
ROC plots, AUC values, 95% confidence interval, p values, cut-off, sensitivity and specificity of miR-122-5p, miR-142-3p, miR-182-5p, miR-183-5p, miR-133a-3p and miR-133b on 70-AC patients (gen+ and gen−) vs. ctrl group.

The identified dysregulated six-miRNA panel was evaluated in the whole cohort of definite AC patients, including both *gen+* and *gen−* AC patients (*n* = 70), by Receiver Operating Characteristic (ROC) curve analysis. Among them, miR-142-3p presented the highest Area Under the Curve (AUC) (0.9692) with a sensitivity of 88.33% and specificity of 100% (cut-off ≤ −0.542), followed by miR-122-5p (AUC = 0.9146, sensitivity of 71.67%, and specificity of 100%) and then miR-133a-3p and miR-133b, with AUC of 0.845 and 0.858, respectively. Instead, miR-182-5p and miR-183-5p showed an AUC of around 0.7 (Figure 3). 

**Figure 4:**
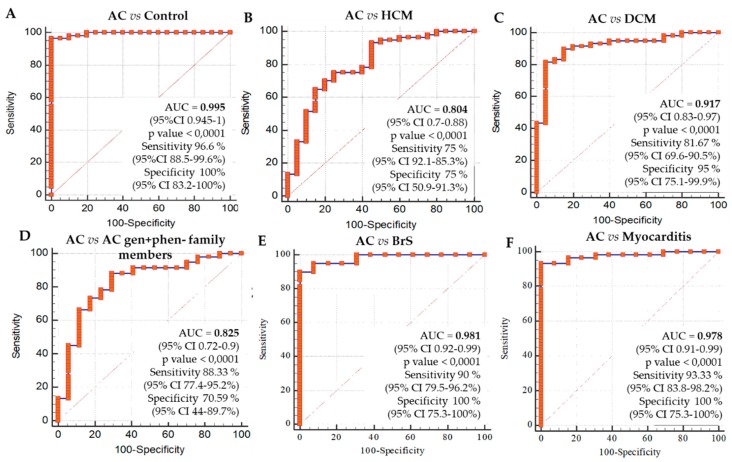
ROC plots, AUC values, p values, sensitivity and specificity of the 6-miRNA panel on: (**A**)—70 AC vs. ctrl; (**B**)—70 AC vs. DCM; (**C**)—70 AC vs. HCM; (**D**)—70 AC vs. AC gen+phen− family members, (**E**)—70 AC vs. BrS; (**F**)—70 AC vs myocarditis.

Multiple logistic regression analysis demonstrated that the entire six-miRNA panel is able to differentiate AC patients when compared to healthy subjects (AUC = 0.995 and a *p* value < 0.001) with a sensitivity of 96.67% and a specificity of 100%. This set of six miRNAs was also able to distinguish patients with ongoing disease from silent family pathogenic-variant carriers, AC *gen+phen−*, with AUC of 0.825 (sensitivity: 88.33% and specificity: 70.59%). Finally, this six-miRNA panel was also able to distinguish AC affected patients from patients with other cardiac entities. The AC group, independent from the genotype, was differentiated from DCM group showing an AUC of 0.917 (sensitivity: 81.67% and specificity: 95%), from the HCM group displaying an AUC of 0.804 (sensitivity: 75% and specificity: 75%), from BrS showing an AUC of 0.981 (sensitivity: 90% and specificity: 100%) and from myocarditis showing an AUC of 0.978 (sensitivity: 93.33% and specificity: 100%) (Figure 4).

### 2.9. In Silico Prediction Analysis

The potential gene targets and signalling pathways were explored for this six-miRNA panel by DIANA mirPath v.3. MiRNA-gene experimentally-supported algorithm, TarBase v7.0 [28], targeted adherens junction (*p* value 1.96 × 10^−7^), Hippo (*p* value 0.002), and TGFβ (*p* value 0.002) signalling pathways. Specifically, the panel targeted 37 genes of the adherens junction pathway, 51 genes of the Hippo signalling pathway, and 33 genes of the TGFβ signalling pathway.

Of note, microT-CDS target prediction algorithm targeted AC pathway predicting 16 genes that were linked to this six-miRNA panel, with a *p* value of 0.02, among which *DSG2* or *JUP* and *TCF7L2*, a transcription factor that is involved in the Wnt/β-catenin pathway [29]. 

Further *in silico* validation analyses showed 1200 possible genes targeted by our miRNAs with experimental evidences of interaction (miRTarBase analysis) and different potentially involved pathways (KEGG analysis). Wnt signalling pathway emerged among the signalling pathways that were correlated to disease pathogenesis with 4.21 × 10^−31^ *p* value. Adherens junction and TGFβ pathway were confirmed with *p* values 2.12 × 10^−8^ and 1.87 × 10^−7^, respectively. Finally, the AC pathway was also targeted, as previously predicted, with a *p* value of 0.0153 showing the interaction of our miRNA panel with 12 genes: *MITF, EP300, ITGB1, BTRC, PTPN1, SMAD4, CCND1, ACTN4, LRP6, MAPK1, ERBB2*, and *CSNK1D*.

## 3. Discussion

MiRNA profiling on myocardial tissue samples of AC patients demonstrated a specific miRNA signature. Six DE miRNAs were in common and validated in a larger AC cohort of blood samples both from Italy and UK, despite differences in the circulating versus myocardial tissue miRNA-expression. This characteristic miRNA AC profile provides non-invasive means to distinguish patients with classic AC phenotype from *gen+* family members without AC phenotype and patients that are affected by other forms of cardiac disease. 

### 3.1. Previous Studies on miRNA in AC

AC is caused by heterozygous pathogenic variants in genes encoding for proteins of the desmosomal complex, identified in nearly 50% of patients and exhibiting low and age-dependent disease penetrance [6]. Thus, it has been advocated that clinical heterogeneity and incomplete penetrance may be caused by other factors than genetics, which synergistically contribute in the expressivity of the disease, such as DE miRNAs [12]. Recent studies in 24 HTx AC patients assessed 1078 miRNAs on myocardial tissue samples and showed that 21 of them were significantly DE, establishing miR-21-5p and miR-135b as the modulators of Wnt and Hippo pathways [22]. Other studies that were performed in PKP2- deficient cardiomyocytes demonstrated that, among 750 miRNAs analysed, only miR-184 downregulation might be implicated in the disease pathogenesis [26]. Instead, transcriptome analysis in cardiac stromal cells derived from three AC patients and three controls showed three DE miRNAs linked to AC, among which miR-29b-3p [25]. Previously, plasma samples of three AC patients and three controls were screened for 377 miRNAs, identifying miR-320a as a possible AC biomarker. The underexpression of miR-320a was then validated in a larger cohort (36 AC, 53 controls, and 21 idiopathic ventricular tachycardia—IVT) showing an AUC of 0.69 [23]. A subsequent study evaluated 11 circulating miRNAs on 28 AC patients with definite diagnosis, 11 with borderline/possible AC diagnosis, and 23 had IVT, showing that four miRNAs (miR-144-3p, miR-145-5p, miR-185-5p, and miR-494) were significantly overexpressed in definite AC patients; specifically, miR-494 showed higher circulating levels in definite AC patients with recurrent ventricular arrhythmias after ablation (AUC = 0.832) [24]. More recently, the miRNA expression profile of a transgenic mouse model with the human DSG2 Q558* truncated protein showed three DE miRNAs when comparing the left ventricle and RV; specifically, miR-217-5p and miR-708-5p were upregulated and miR-499-5p were downregulated in the mouse RV [30]. 

Taken together, literature and data from our experiments on myocardial tissue and blood samples that were derived from AC patients, tissue-related miRNA expression profiling showed miR-21-5p, miR-29b-3p, and miR-144-3p overexpression, which was not observed in blood samples from AC patients. Similarly, miR-320a and miR-494 were significantly underexpressed in the myocardium, but no reduction of circulating levels was detected in the larger AC cohort. The other previously reported miRNAs were either normally expressed in tissue and in circulation or they were not detected by small RNA-Seq (miR-184, miR-135b, miR-145-5p, miR-185-5p, miR-217-5p, miR-708-5p, and miR-499-5p). The above findings demonstrated that the use of different technical approaches, analysis strategies, and specimens (animal models, cells, plasma) for miRNA detection, may deeply influence the congruence among studies. 

On the contrary, our miRNA expression profiles that were obtained from the RV of HTx AC patients and blood samples from AC patients fulfilling TFC showed an unbiased six DE miRNAs signature obtained after a thoughtful experimental design and deep filtering of miRNAs through the validation process from a wide microRNA screening with two different techniques to the six-miRNA panel validated in the largest cohort reported so far. We performed our analysis, including only AC patients with definite TFC, as demonstrated by the same miRNA profile that was obtained both in *gen+* and *gen−* patients as well as an independent AC-UK cohort with *gen+* and *gen−* patients. This miRNA profile is independent from the genotype and it is likely a consequence of the disease phenotype. 

Further, AC-related miRNA signature specificity was supported by an *in silico* correlation with signalling pathways that were linked to disease pathogenesis. Based on the target microT-CDS predictor, our six miRNA panel directly targeted 16 genes that were related to AC, among which the components of the desmosomal complex or transcription factors ie *JUP*, *DSG2*, or *TCF7L2*. Additionally, the experimentally validated miRNA-gene interactions showed a correlation with Wnt, Hippo, and/or TGFβ signalling pathways [31,32]. However, other studies will be necessary to determine the role of these six DE miRNAs as the precursors or consequence of the disease phenotype. 

### 3.2. Comparison between Tissue and Blood miRNA Profiles

Several studies advanced a possible role of circulating miRNAs in distant cell-cell communication through their secretion into protective vesicles (exosomes), bound to RNA-binding proteins, or even released within apoptotic bodies [12]. In an acute myocardial infarction model, it was demonstrated that increased plasma miRNA levels were the consequence of myocardial cell damage [33], and in a liver injury model, decreased hepatic levels of miRNAs reflected their increase in patients’ plasma as if they were released from the injured liver to circulation [34]. Further, the miRNA expression profiles in tumor cells demonstrated the release of these miRNAs from the tissue in the blood, highlighting that circulating DE miRNAs may be the consequence of tumour tissue changes detectable in blood as non-invasive biomarkers [35]. 

Accordingly, four of our six miRNA-panel lacked directionality consistency in tissue and blood profiles. Specifically, we observed three miRNAs (miR-122-5p, miR-182-5p, miR-182-3p) that were underexpressed in tissue and overexpressed in the circulation, supporting the hypothesis that the high blood levels of miRNAs might be released from apoptotic or necrotic cardiomyocytes, indicating progression of tissue damage. As such, we presume that myocardial damage is reflected in the circulation and that these miRNAs can be used as non-invasive biomarkers of the disease, enabling differential diagnosis. Whether the release of these specific miRNAs in the circulation is due to active secretion by macrovesicles or passive release/leakage by apoptotic cardiomyocytes needs to be elucidated. 

On the other hand, miRNA overexpression in tissue and underexpression in the circulation might indicate an active uptake of miRNAs by different cell types, as demonstrated in Acute Myeloid Leukemia, in which the miR-92a levels were decreased in plasma and increased in cells [36]. As such, we found miR-142-3p overexpression in myocardial tissue and underexpression in circulation, supporting the idea of miRNAs active uptake from the bloodstream into the myocardial tissue. Additional studies should determine the cell origin of miR-142-3p and its role in disease pathogenesis.

### 3.3. Discriminatory Power: A New Biomarker for AC?

Current diagnosis rests on fulfilling a set of clinical criteria and on genetic testing, which are relatively specific, but not highly sensitive. In the largest AC cohort with definite TFC so far, we demonstrated, by a comprehensive miRNA expression analysis, that the expression level of six DE miRNAs is a consistent feature in patients with AC, and it is not found in other forms of heart-muscle disease. The strength of this six-miRNA signature is based on its ability to differentiate patients affected by AC from healthy subjects with approximately 97% sensibility and 100% specificity. In this study, *gen+* and *gen−* AC TFC patients from Padua and UCL research centers shared the same miRNA profile as to indicate that expression alterations might be a consequence of the disease phenotype. The lack of known biomarkers in AC makes its validation to be used as biomarker difficult and challenging.

Further, the same six-miRNA dataset differentiated AC index cases from patients affected by other primary cardiomyopathies or phenocopies, such as DCM, HCM, BrS, and myocarditis. In this setting, the AC patients were discerned from DCM patients with 82% sensitivity and 95% specificity, from HCM patients with 75% sensitivity and 75% specificity, from BrS patients with 90% sensitivity and 100% specificity, and from myocarditis patients with 93% sensitivity and 100% specificity. Their ability as a whole set to differentiate the disease phenotype from other cardiac diseases led to the hypothesis that this six miRNA signature might be used as a non-invasive biomarker of the disease. Although additional validation studies will be required before this new test can be used clinically, this is a potentially important distinction, which hints at disease-specific mechanisms and suggests that DE miRNAs may cause clinical presentation variability. Finally, this potential biomarker was able to differentiate AC patients with disease phenotype from AC *gen+phen−* family members, so as to underline the fact that the genetic status alone is not sufficient to determine fully penetrant disease. As such, this six-miRNA set is a potential tool for establishing AC diagnosis in family members of an index patient without extensive clinical workup, which often leads to equivocal results. A longer follow up period and a larger cohort are needed to demonstrate its predictive value for disease prognosis.

## 4. Materials and Methods

### 4.1. Study Cohort

The study population (discovery and validation cohorts, Figure 1A) included 106 unrelated AC index patients, without cardiac or extracardiac major comorbidities, according to revised criteria established by 2010 Task Force (TFC): 86 from Padua (AC-PD Supplementary Material Table S1, #AC1–AC16, #1–70) and 20 from the University College of London-UCL (United Kingdom, AC-UK) [3], 28 healthy *ctrl* and an independent validation cohort comprising: 17 non-affected family members of AC probands carrying a desmosomal pathogenic variant (*AC gen+phen−*), 20 HCM, 20 DCM, 13 BrS and 13 myocarditis affected patients. 

In this regard, we analysed nine frozen right ventricle (RV) myocardial samples from heart transplanted (HTx, disease end-stage) AC index cases (AC1–AC9) and 99 whole blood samples of AC definite TFC patients in both discovery and validation cohorts (AC1, AC2, AC10–AC16, #1–70, 20 AC-UK, Figure 1A). HTx AC cases presented an end-stage disease with biventricular involvement. The 99 blood samples were collected from index cases with a definite AC diagnosis according to TFC that was mostly enrolled for arrhythmic clinical presentation and without heart failure symptoms. Exception made for 2 HTx patients (AC1, AC2) who underwent analysis for both blood and heart tissue specimens. The *ctrl* group comprised four RV myocardial tissue samples from subjects who died from conditions other than cardiac diseases and 24 blood samples of healthy subjects. Finally, only whole blood samples were analysed in the independent validation cohort (Figure 1A). HCM, DCM, and BrS affected patients were diagnosed according to the current recommendations for each cardiac disease [37,38,39] based on their symptoms and clinical signs, while myocarditis affected patients were diagnosed by endomyocardial biopsy [40].

Next Generation Sequencing previously screened the entire study population (TruSight Cardio Sequencing Kit, Illumina, San Diego, CA, USA) and written informed consent was provided for all samples, conforming to the principles that were outlined in the Declaration of Helsinki. In particular, 62 of the 86 AC-PD carried a rare variant on a desmosomal gene (AC *gen+*) and 24 were genotype-negative (AC *gen−*) (Table 1, Supplementary Material Table S1). Ten of 20 AC-UK index cases carried at least a rare variant on a desmosomal gene: 5 patients carried a rare variant in *DSP*, 3 in *PKP2* and 2 were compound heterozygous, *DSP/PKP2* and *PKP2/JUP*. All 20 HCM, 20 DCM, and 13 BrS index cases carried a rare variant in a disease-associated gene and 13 myocarditis cases were free of pathogenic variants in cardiac-related genes. The variants were classified according to American College of American Genetics (ACMG) [26]. 

### 4.2. miRNA Isolation and Quantification

Total RNA, including small RNAs, was isolated from 20 mg frozen RV myocardial tissue and 50 µL of whole blood samples while using RNeasy Mini Kit (Qiagen, Hilden, Germany), according to manufacturer’s instructions (Supplementary Material).

### 4.3. miRNA Screening

84 miRNA cardiac-related array analysis was performed on LightCycler 480 platform (Roche, Basel, Switzerland ) for tissue (AC1–AC9) samples and blood (AC1, AC2, AC10–AC16) samples from nine AC patients and 4 *ctrl*, respectively, while using MiScript miRNA PCR Array Human Cardiovascular disease (Qiagen, Hilden, Germany), according to the manufacturer’s instructions (Supplementary Material). Data analysis and normalization were performed with the GeneGlobe analysis tool (Qiagen, Hilden, Germany). 

Library preparation while using TruSeq Small RNA Library Preparation kit (Illumina, San Diego, CA, USA) was used on tissue samples of three AC (AC1–AC3) probands and two *ctrl* whereas Next Flex Small RNASeq kit (PerkinElmer, Waltham, MA, USA was used on blood samples of 4 AC (AC1, AC2, AC10–11) probands and 4 *ctrl,* according to manufacturer’s instructions. Equimolar pooled samples (10pM) were sequenced on a MiSeq platform (Illumina, San Diego, CA, USA). BaseSpace Sequence Hub was used for alignment and subsequent pairwise differential expression analysis (DESeq2) and it led to the identification of DE miRNAs, precursor groups, miRNAs families, and piRNAs for each pair of sample groups (Supplementary Material). 

### 4.4. Quantitative PCR Validation

DE miRNAs were validated by quantitative real-time Polymerase Chain Reaction (qPCR) on LightCycler 480 platform (Roche, Basel, Switzerland). Relative Quantification Analysis was carried out by ΔΔ*C*t method with *C. elegans* miR-39 as spike-in *ctrl* to normalize all data (Supplementary Material).

### 4.5. In Silico Target Prediction

*In silico* target prediction was performed while using DIANA mirPath v3 [28], a web-miRNA targets prediction server based on experimentally validated (TarBase v7.0) and theoretical (microT-CDS) miRNA:gene interactions, providing pathways that were enriched with targeted genes of DE miRNAs. Additionally, miRTarBase v2018 [30] was interrogated for experimentally validated targeted genes of miRNAs, and they were subsequently analysed on NetworkAnalyst. 

### 4.6. Statistical Analysis

Statistical analysis was conducted with GraphPad Prism version 7.0 for Windows (GraphPad Software, La Jolla, CA, USA, www.graphpad.com) and MedCalc Statistical Software version 16.4.3 (MedCalc Software bvba, Ostend, Belgium; https://www.medcalc.org; 2016). Continuous variables are always presented as mean ± standard error of mean (mean ± SEM), unless otherwise specified. The results are expressed in terms of Log_2_Fold-change (Log_2_FC). A miRNA was considered to be underexpressed when the fold-change is lower than 0.5 (Log_2_FC < −1) and overexpressed when the fold-change is greater than 2 (Log_2_FC > 1).

The expression of each miRNA was compared between the disease-groups and a control group with non-parametric Mann-Whitney test since the distribution was not normal and Kruskal-Wallis test was used for multiple comparisons adjusting the *p* value with the False Discovery Rate method (two-stage step-up Benjamini, Krieger, and Yekutieli). 

The area under the curve (AUC) of Receiver Operating Characteristic (ROC) plots are two-dimensional graphs, in which sensitivity is plotted on the Y axis and 1-specificity is plotted on the X axis. The closer the apex of the curve toward the upper left corner, the greater the discriminatory ability of the test (i.e., the true-positive rate is high and the false-positive 1–Specificity rate is low). This is quantitatively measured by the AUC. The maximum value for the AUC is 1.0, thereby indicating a (theoretically) perfect test (i.e., 100% sensitive and 100% specific). An AUC value of 0.5 indicates no discriminative value (i.e., 50% sensitive and 50% specific) and it is represented by a straight, diagonal line extending from the lower left corner to the upper right. There are several scales for AUC value interpretation, but, in general, ROC curves with an AUC ≤0.7 are not clinically useful and an AUC of 0.97 has very high clinical value. However, there are confidence intervals around this value that must be taken into consideration [41]. AUC was estimated with 95% confidence interval to assess the diagnostic accuracy of each selected miRNA. Sensitivity and specificity were estimated with a 95% confidence interval after detecting the cut-off value with the Youden method. Multiple logistic regression analysis was performed on the six miRNAs from the final panel to study the diagnostic power of all siz miRNAs together on AC (*gen+, gen−*) group in comparison with *ctrl*, AC *gen+phen−*, HCM, DCM, BrS and Myocarditis groups. ROC analysis was performed on predicted results.

## 5. Conclusions

Our miRNA expression profiles that were obtained from the RV of HTx AC patients and blood samples from AC patients fulfilling TFC, showed an unbiased six DE miRNAs signature identified while using a targeted experimental design and a deep filtering validation process. 

The unique six-miRNA panel identified in this study is characteristic and specific for AC, exhibiting a great discriminatory diagnostic power. In this setting, this set of miRNAs is the best potential miRNA panel as non-invasive AC diagnostic biomarker reported so far.

## 6. Limitations

In this study, blood and tissue samples from a small AC cohort of genotype-positive index cases were used as a discovery cohort to identify a subset of miRNAs in the blood helpful for the detection of AC in a non-invasive way. In this setting, more miRNAs were DE using Small RNA-seq, which have not been further investigated. The largest AC cohort reported so far was used for validation of this subset of miRNAs to overcome the bias of the small discovery cohort. Although several independent validation cohorts (*gen+phen−* family members and different cardiac-disease groups) were used to verify the specificity and sensitivity of our data, the number of subjects included in each cohort is small. Larger cardiac-disease cohorts are required to determine the possible clinical use of this miRNA set. Further experiments on targeted signaling pathways by these miRNAs and gene expression analysis are necessary for determining their implication in disease pathogenesis. 

## Figures and Tables

**Table 1:** Discovery cohort: genetics and clinical data.

Sample ID	Specimen	AC TFC Criteria	Gene	cDNA	Protein	dbSNP ID	MAF gnomAD	ACMG Variant Classification
AC 1	RV myocardial tissue	2M	*PKP2*	c.1643delG	p.Gly548ValfsX15	rs794729137	0.00002	Pathogenic
AC 2	RV myocardial tissue	3M	*PKP2*	c.2013delC	p.Lys672ArgfsX12	rs764817683	0.0000081	Pathogenic
AC 3	RV myocardial tissue	1M, 2m	*PKP2*	chr12:32,782,127-133,275,309 heterozygous deletion				Pathogenic
AC 4	RV myocardial tissue	2M	*DSP*	c.2956C > T	p.Gln986X	NA	NA	Pathogenic
AC 5	RV myocardial tissue	4M	*DSP*	c.5644G > T	p.Glu1882X	NA	NA	Pathogenic
AC 6	RV myocardial tissue	3M, 1m	*DSP*	c.5511dupT	p.Arg1838SerfsX19	NA	NA	Pathogenic
AC 7	RV myocardial tissue	2M	*DSG2*	c.797A > G	p.Asn266Ser	rs121913011	0.0000041	Pathogenic
AC 8	RV myocardial tissue	2M	*DSG2*	c.1672C > T	p.Gln558X	NA	NA	Pathogenic
AC 9	RV myocardial tissue	1M, 2m	*DSG2*	c.2033G > C	p.Gly678Ala	rs372494397	0.00004	VUS *
AC 1	Whole blood	2M	*PKP2*	c.1643delG	p.Gly548ValfsX15	rs794729137	0.00002	Pathogenic
AC 2	Whole blood	3M	*PKP2*	c.2013delC	p.Lys672ArgfsX12	rs764817683	0.0000081	Pathogenic
AC 10	Whole blood	3M, 1m	*PKP2*	c.631C > T	p.Gln211X	NA	NA	Pathogenic
AC 11	Whole blood	3M	*PKP2*	c.2013delC	p.Lys672ArgfsX12	rs764817683	0.0000081	Pathogenic
AC 12	Whole blood	2M, 2m	*PKP2*	c.964_965delinsC	p.Gly322ProfsX30	NA	NA	Likely pathogenic
AC 13	Whole blood	3M	*DSG2*	c.797A > G	p.Asn266Ser	rs121913011	0.0000041	Pathogenic
AC 14	Whole blood	3M	*DSP*	c.3889C > T	p.Gln1297X	NA	0.0000041	Pathogenic
AC 15	Whole blood	1M, 2m	*DSG2/ DSC2*	chr18:31,065,974-31,549,007 heterozygous deletion				Pathogenic
AC 16	Whole blood	2M, 1m	*DSP*	c.337C > T	p.Gln113X	NA	NA	Pathogenic
			*DSG2*	c.797A > G	p.Asn266Ser	rs121913011	0.0000041	Pathogenic

Characteristics of variants in AC *gen+* index cases (discovery cohort) used for miRNA screening analysis. MAF: Minor Allele Frequency. Major (M) and minor (m) criteria according to Task Force Criteria (TFC) of AC [3]. Variant classification according to American College of Medical Genetics (ACMG) guidelines [26]. * co-segregation with disease phenotype within the family.

**Table 2:** Six-miRNA panel expression assessed on validation and independent validation cohorts.

Validation and Independent Validation Cohorts						
Log_2_FC (mean ±SEM) *p* value (Mann-Whitney test) *p* value* (Kruskal-Wallis adjusted FDR)						
	miR-122-5p	miR-142-3p	miR-182-5p	miR-183-5p	miR-133a-3p	miR-133b
AC *gen+*	1.87 ± 0.20	−2.4 ± 0.35	0.78 ± 0.19	0.71 ± 0.12	−1.15 ± 0.27	−1.29 ± 0.22
	*p* < 0.0001	*p* < 0.0001	*p* = 0.0002	*p* < 0.0001	*p* < 0.0001	*p* < 0.0001
	p* < 0.0001	p* < 0.0001	p* = 0.0045	p* = 0.0161	p* = 0.0081	p* = 0.0007
AC *gen−*	3.20 ± 0.64	−4.23 ± 0.47	0.82 ± 0.24	1.16 ± 0.26	−2.44 ± 0.51	−1.75 ± 0.57
	*p* < 0.0001	*p* < 0.0001	*p* = 0.0096	*p* = 0.0009	*p* < 0.0001	*p* = 0.0002
	p* < 0.0001	p* < 0.0001	p* = 0.0170	p* = 0.0029	p* < 0.0001	p* < 0.0001
AC-UK	2.34 ± 0.49	−8.54 ± 0.57	1.05 ± 0.13	1.04 ± 0.28	−2.91 ± 0.59	−1.63 ± 0.42
	*p* < 0.0001	*p* < 0.0001	*p* < 0.0001	*p* = 0.001	*p* < 0.0001	*p* < 0.0001
	p* < 0.0001	p* < 0.0001	p* = 0.0021	p* = 0.0074	p* < 0.0001	p* = 0.0007
AC *gen + phen−*	0.67 ± 0.32	−2.88 ± 0.25	−0.17 ± 0.28	−0.27 ± 0.25	−2.62 ± 0.30	−2.27 ± 0.30
	*p* = 0.063	*p* < 0.0001	*p* = 0.269	*p* = 0.060	*p* < 0.0001	*p* < 0.0001
	p* = 0.1458	p* = 0.0001	p* = 0.7852	p* = 0.4724	p* < 0.0001	p* < 0.0001
HCM	1.98 ± 0.56	−0.87 ± 0.07	0.72 ± 0.49	0.72 ± 0.43	−0.58 ± 0.38	−0.62 ± 0.34
	*p* = 0.002	*p* = 0.073	*p* = 0.049	*p* = 0.026	*p* = 0.217	*p* = 0.465
	p* = 0.0001	p* = 0.2599	p* = 0.0221	p* = 0.0219	p* = 0.1753	p* = 0.1388
DCM	1.45 ± 0.31	−5.49 ± 0.66	−0.83 ± 0.32	−0.36 ± 0.30	−2.90 ± 0.37	−3.01 ± 0.32
	*p* < 0.0001	*p* < 0.0001	*p* = 0.085	*p* = 0.792	*p* < 0.0001	*p* < 0.0001
	p* = 0.0021	p* < 0.0001	p* = 0.2373	p* = 0.8852	p* < 0.0001	p* < 0.0001
BrS	2.84 ± 1.306	−6.58 ± 0.59	−1.27 ± 0.50	−0.31 ± 0.40	−4.38 ± 0.45	−3.24 ± 0.51
	*p* = 0.057	*p* < 0.0001	*p* = 0.0087	*p* = 0.2829	*p* < 0.0001	*p* < 0.0001
	p* = 0.0037	p* < 0.0001	p* = 0.1689	p* = 0.6971	p* < 0.0001	p* < 0.0001
Myocarditis	4.57 ± 1.47	−7.11 ± 0.48	−1.38 ± 0.42	−0.34 ± 0.39	−1.61 ± 1.17	−1.39 ± 1.21
	*p* = 0.0001	*p* < 0.0001	*p* = 0.0003	*p* = 0.889	*p* = 0.070	*p* = 0.125
	p* < 0.0001	p* < 0.0001	p* = 0.0748	p* = 0.7245	p* = 0.0013	p* = 0.0042

Log_2_FC (mean ±SEM) values of the 6 miRNA-panel in AC *gen+*, AC *gen−* and AC-UK (validation cohorts); and AC *gen+phen−*, HCM, DCM, BrS, Myocarditis groups (independent validation cohorts) compared to the *ctrl* group; *p* value (p) calculated by Mann-Whitney test when compared separately, *p* values (p*) calculated by Kruskal-Wallis for multiple comparison adjusted by False Discovery Rate two-stage step-up method for Benjamini, Krieger, and Yekutieli.

## Supplementary Materials

The following are available online at https://www.mdpi.com/1422-0067/21/4/1536/s1, Figure S1: DE miRNAs in the AC-tissue profile and blood profile on the discovery cohort by the 84 cardiac-related array (Log_2_FC, mean ±SEM). Figure S2: Gene-dependent miRNA analysis. A: Clustergram of the 84 cardiac-related array analysis on the nine samples divided based on mutated gene; group 1—PKP2, group 2—DSP, group 3—DSG2. B: DE miRNAs of PKP2 profile. C: DE miRNAs of DSP profile. D: DE miRNAs of DSG2 profile; Log_2_FC (mean ±SEM). Table S1: Clinical data of the AC index cases including discovery and validation cohorts. Table S2: Discovery cohort DE miRNAs shared between tissue and blood arrays; Log_2_FC (mean ±SEM) values of the 10 miRNAs assessed by 84 cardiac-related array. Consistent directionality of the DE in tissue versus blood. Table S3: Thirteen miRNAs expression in the validation cohort; Log_2_FC (mean ±SEM) values of the 13 miRNA analysed on 46 AC gen+ and 20 controls (validation cohort); p value calculated by the Mann-Whitney test.
