# Supplementary material for: A microRNA Expression Profile as Non-Invasive Biomarker in a Large Arrhythmogenic Cardiomyopathy Cohort
Source: Int J Mol Sci. 2020 Feb 24;21(4):1536. doi: 10.3390/ijms21041536 (PMC7073183; doi:10.3390/ijms21041536)
Supplement: Supplementary file 1 [file ijms-21-01536-s001.zip › Supplementary Materail.docx]

A microRNA expression profile as non-invasive biomarker in a large Arrhythmogenic Cardiomyopathy cohort.

**Maria Bueno Marinas, PhD^1^, Rudy Celeghin, PhD^1^, Marco Cason, PhD^1^, Riccardo Bariani, MD^1^, Anna Chiara Frigo, PhD^1^, Joanna Jager, MSc^2^, Petros Syrris, PhD^2^, Perry Elliott, MD^2^, Barbara Bauce, MD, PhD^1^, Gaetano Thiene, MD^1^, Domenico Corrado, MD, PhD^1^, Cristina Basso, MD, PhD^1^, Kalliopi Pilichou, PhD^1^.**

1. Department of Cardiac-Thoracic-Vascular Sciences and Public Health, University of Padua, Cardiovascular Pathology, Cardiology and Biostatistics Units, Padua, Italy.
2. Centre for Heart Muscle Disease, Institute of Cardiovascular Science, University College London, London, UK

***** Correspondence:

Kalliopi Pilichou, PhD

Cardiovascular Pathology Unit

Department of Cardiac-Thoracic-Vascular Sciences and Public Health,

University of Padova- Azienda Ospedaliera di Padova

Via A. Gabelli, 61-35121 Padova Italy

kalliopi.pilichou@unipd.it; Tel.: +39 0498272293

**Supplemental Material and methods**

1. *MiRNA isolation and reverse transcription*

Total RNA, including small RNAs, was isolated from 20 mg frozen RV myocardial tissue samples with RNeasy Mini Kit (Qiagen, Germany) according to manufacturer’s instructions modifying the percentage of ethanol in alcohol precipitation step to 100% and adding a clean-up step after RNA isolation. Instead, circulating RNA was isolated from 50 µl of frozen whole blood with MagMAX mirVana Total RNA Isolation Kit (Thermo Fisher Scientific, USA) with the addition of a spike-in control *C. elegans* miR-39, miRNA mimic (Qiagen, Germany).

Reverse transcription was performed on 100 ng of total RNA using miScript II RT kit (Qiagen, Germany), where it is only reverse transcribed the small RNA fraction specifically, following manufacturer’s instructions.

1. *RNA and miRNA quantification*

Spectrophotometric and fluorimetric methods were used to quantify total RNA, in particular Nanovue Spectophotometer (GE Healthcare Life Sciences, USA) and Qubit (Thermo Fisher Scientific, USA). In addition, small RNA kit for Qubit was used to measure only microRNA. For NGS application, RNA quality was assessed using Agilent 2100 Bioanalyzer system (Agilent, USA).

1. *84 miRNA cardiac-related array analysis*

cDNA obtained from RV myocardial tissue samples of 9 AC patients and 4 ctrl, and from blood samples of 9 AC patients and 4 ctrl underwent miRNA screening with miScript miRNA PCR Array Human Cardiovascular disease (Qiagen, Germany) following user’s manual on LightCycler 480 platform (Roche, Germany); consisting in an array with 84 miRNAs correlated with cardiovascular diseases, 6 housekeeping small RNAs and two positive controls. Ct values collection was performed using Second Derivative Maximum analysis method. Ct, cycle threshold, is defined as the number of cycles required for the fluorescent signal to cross the threshold and were subsequently analysed by Qiagen Data Analysis Center.

1. *Small RNA Sequencing*

Final libraries (tissue and blood libraries) were loaded separately in a MiSeq Reagent kit v3 and 75bp RNA single-reads were sequenced (Small RNA-seq) on MiSeq platform. FASTQ files were analysed on BaseSpace with Small RNA application v.1.0.1 which aligns reads (Botwie v0.12.8) to four reference databases (contaminants, mature miRNAs, RNA and reference genome) and output reads to mature miRNAs, isomiRs and PIWI-interacting RNAs (piRNAs).

1. *Quantitative PCR Validation*

The DE miRNAs were further validated by quantitative real-time Polymerase Chain Reaction (qPCR) on LightCycler 480 platform (Roche, Germany). Briefly, all samples were tested in triplicate using 1 µl of cDNA obtained by reverse transcription with miScript II RT kit (Qiagen, Germany), specific forward primer with final concentration of 300 nM, and miScript SYBR Green PCR kit (Qiagen, Germany), following user’s manual. Specific forward primers were designed using miRprimer software 31. Instead, for miR-133a-3p, miR-133b and cel-miR-39, miScript primer assays (Qiagen, Germany) were used following user’s manual. The spike-in control *C. elegans* miR-39 was used to normalize all data. Relative Quantification Analysis was carried out by ΔΔCt method.

1. *In silico target prediction*

*In silico* target prediction was performed using DIANA mirPath v3 [1], consisting in a web-server that provides accurate statistics for predicted miRNA targets based on experimentally validated miRNA:gene interactions (TarBase v7.0 [2]); and predicted miRNA:gene interactions (microT-CDS [3]), providing pathways enriched with genes belonging the union of targeted genes of differentially expressed miRNAs by KEGG analysis. Additionally, miRTarBase v 2018 [4] was interrogated for experimentally validated targeted genes of miRNAs, and were subsequently analysed on NetworkAnalyst [5]

.

**Supplemental Tables**

Table S1. Clinical data of the AC index cases including discovery and validation cohorts.

| **Case number (#)** | **Sex** | **Age (yrs) at diagnosis/ last investigation** | **I. Global or regional dysfunction and structural alterations** | | **II. Tissue characterization of wall** | **III. Repolarization abnormalities** | | | **IV.Depolarization/ conduction abnormalities** | | **V.Arrhythmias** | | | **VI. Family history** | | **LE-CMR** | **AC diagnostic criteria** | **Gene** |
| --- | --- | --- | --- | --- | --- | --- | --- | --- | --- | --- | --- | --- | --- | --- | --- | --- | --- | --- |
|  |  |  | **Echocardiography** | |  | **Twelve-lead ECG** | | | **Twelve-lead ECG** | | **Holter ECG** | | |  |  |  |  |  |
|  |  |  | Regional RV akinesia, dyskinesia, or aneurysm and 1 of the following (end diastole): | |  |  |  |  |  |  |  |  |  |  |  |  |  |  |
|  |  |  | PLAX RVOT ≥32 mm; PSAX RVOT ≥36 mm; FAC ≤33% (**M**) | PLAX RVOT ≥29 to <32 mm; PSAX RVOT ≥32 to <36 mm; FAC >33% to ≤40% (**m**) |  | Inverted T waves in V1-V3 (>14 yrs) (**M**) | Inverted T waves in V1-V2 (>14 yrs) or V4-V6 (**m**) | Inverted T waves in V1-V4 (>14 yrs) in presence of RBBB (**m**) | Epsilon wave (**M**) | Late potentials (SAECG) (**m**) | NSVT or SVT of LBBB morphology with superior axis (**M**) | NSVT or SVT of RV outflow configuration or of unknown axis (**m**) | >500 ventricular extrasistoles per 24 hours (holter) (**m**) | (**M**) | (**m**) |  |  |  |
| AC1 | M | 70 | 1 | 0 | 1 | 0 | 0 | 0 | 0 | 0 | 0 | 0 | 0 | 0 | 0 | NA | 2M | PKP2 |
| AC2 | M | 30 | 0 | 0 | 1 | 1 | 0 | 0 | 0 | 0 | 1 | 0 | 0 | 0 | 0 | NA | 3M | PKP2 |
| AC3 | F | 44 | 1 | 0 | 0 | 0 | 0 | 1 | 0 | 1 | 0 | 0 | 0 | 0 | 0 | 1 | 1M, 2m | PKP2 |
| AC4 | F | 60 | 1 | 0 | 1 | 0 | 0 | 0 | 0 | 0 | 0 | 0 | 0 | 0 | 0 | NA | 2M | DSP |
| AC5 | F | 51 | 1 | 0 | 1 | 0 | 0 | 0 | 0 | 0 | 1 | 0 | 0 | 1 | 0 | NA | 4M | DSP |
| AC6 | M | 66 | 1 | 0 | 1 | 0 | 1 | 0 | 0 | 0 | 1 | 0 | 0 | 0 | 0 | NA | 3M, 1m | DSP |
| AC7 | F | 65 | 1 | 0 | 1 | 0 | 0 | 0 | 0 | 0 | 0 | 0 | 0 | 0 | 0 | NA | 2M | DSG2 |
| AC8 | F | 47 | 1 | 0 | 1 | 0 | 0 | 0 | 0 | 0 | 0 | 0 | 0 | 0 | 0 | NA | 2M | DSG2 |
| AC9 | F | 63 | 0 | 0 | 1 | 0 | 0 | 1 | 0 | 1 | 0 | 0 | 0 | 0 | 0 | 1 | 1M, 2m | DSG2 |
| AC10 | F | 16 | 1 | 0 | 0 | 1 | 0 | 0 | 0 | 1 | 1 | 0 | 0 | 0 | 0 | 1 | 3M, 1m | PKP2 |
| AC11 | M | 49 | 1 | 0 | 0 | 1 | 0 | 0 | 1 | 0 | 0 | 0 | 0 | 0 | 0 | NA | 3M | PKP2 |
| AC12 | F | 39 | 1 | 0 | 0 | 1 | 0 | 0 | 0 | 1 | 0 | 0 | 1 | 0 | 0 | NA | 2M, 2m | PKP2 |
| AC13 | F | 40 | 1 | 0 | 0 | 0 | 0 | 0 | 0 | 0 | 1 | 0 | 0 | 1 | 0 | NA | 3M | DSG2 |
| AC14 | F | 52 | 1 | 0 | 0 | 0 | 0 | 0 | 0 | 0 | 1 | 0 | 0 | 1 | 0 | 1 | 3M | DSP |
| AC15 | M | 14 | 0 | 1 | 0 | 1 | 0 | 0 | 0 | 1 | 0 | 0 | 0 | 0 | 0 | 1 | 1M, 2m | DSG2/DSC2 |
| AC16 | M | 69 | 1 | 0 | 0 | 1 | 0 | 0 | 0 | 1 | 0 | 0 | 0 | 0 | 0 | NA | 2M, 1m | DSG2/DSP |
| 1 | M | 39 | 1 | 0 | 0 | 0 | 0 | 0 | 1 | 0 | 0 | 0 | 0 | 0 | 0 | NA | 2M | DSP |
| 2 | F | 47 | 0 | 1 | 0 | 1 | 0 | 0 | 0 | 1 | 0 | 0 | 1 | 0 | 0 | 0 | 1 M 3m | PKP2 |
| 3 | F | 26 | 0 | 1 | 1 | 1 | 0 | 0 | 0 | 0 | 0 | 0 | 0 | 0 | 0 | 1 | 2M, 1m | PKP2 |
| 4 | F | 40 | 1 | 0 | 1 | 1 | 0 | 0 | 0 | 1 | 0 | 1 | 0 | 0 | 0 | NA | 3M, 2m | PKP2 |
| 5 | F | 58 | 1 | 0 | 0 | 1 | 0 | 0 | 0 | 0 | 0 | 0 | 1 | 0 | 0 | NA | 2M, 1m | DSC2 |
| 6 | F | 61 | 1 | 0 | 0 | 1 | 0 | 0 | 0 | 0 | 0 | 1 | 0 | 0 | 0 | NA | 2M, 1m | DSG2/PKP2 |
| 7 | M | 16 | 1 | 0 | 1 | 0 | 0 | 0 | 0 | 0 | 0 | 0 | 0 | 0 | 0 | 1 | 2M | DSG2/PKP2 |
| 8 | M | 57 | 1 | 1 | 0 | 0 | 0 | 0 | 0 | 1 | 0 | 0 | 0 | 0 | 0 | NA | 1 M 2m | DSC2 |
| 9 | M | 45 | 1 | 0 | 0 | 1 | 0 | 0 | 0 | 1 | 0 | 0 | 1 | 0 |  | NA | 2M, 2m | DSC2 |
| 10 | M | 42 | 0 | 0 | 0 | 1 | 0 | 0 | 0 | 0 | 1 | 0 | 0 | 0 | 0 | NA | 2M | DSG2 |
| 11 | M | 11 | 1 | 1 | 0 | 0 | 0 | 0 | 0 | 0 | 1 | 0 | 0 | 0 | 0 | NA | 3M | PKP2 |
| 12 | M | 10 | 1 | 0 | 0 | 1 | 0 | 0 | 0 | 1 | 0 | 0 | 0 | 0 | 0 | 1 | 2M. 1m | DSG2 |
| 13 | F | 59 | 0 | 0 | 0 | 1 | 0 | 0 | 0 | 1 | 0 | 0 | 1 | 0 | 0 | 0 | 1M, 2m | PKP2 |
| 14 | F | 50 | 0 | 1 | 1 | 1 | 0 | 0 | 0 | 0 | 0 | 1 | 0 | 0 | 0 | 0 | 2M, 2m | PKP2 |
| 15 | M | 60 | 1 | 0 | 0 | 0 | 1 | 0 | 0 | 0 | 0 | 0 | 1 | 0 | 0 | 1 | 1M, 2m | DSG2 |
| 16 | F | 41 | 1 | 0 | 1 | 0 | 0 | 0 | 0 | 0 | 0 | 0 | 0 | 0 | 0 | NA | 2M | DSP |
| 17 | M | 37 | 1 | 0 | 0 | 1 | 0 | 0 | 0 | 0 | 0 | 0 | 0 | 0 | 0 | 1 | 2M | DSC2 |
| 18 | M | 49 | 1 | 0 | 0 | 1 | 0 | 0 | 1 | 0 | 0 |  | 0 | 0 | 0 | 0 | 3M | PKP2 |
| 19 | F | 32 | 1 | 0 | 1 | 0 | 0 | 0 | 0 | 0 | 0 | 0 | 0 | 0 | 0 | NA | 2M | DSP/PKP2 |
| 20 | M | 19 | 1 | 0 | 1 | 0 | 0 | 0 | 0 | 0 | 0 | 0 | 0 | 0 | 0 | NA | 2M | DSG2/PKP2 |
| 21 | F | 67 | 0 | 0 | 0 | 1 | 0 | 0 | 0 | 1 | 0 | 0 | 1 | 0 | 0 | NA | 1M, 2m | DSP |
| 22 | F | 37 | 0 | 0 | 1 | 0 | 0 | 0 | 0 | 1 | 0 | 0 | 0 | 1 | 0 | 0 | 2M, 1m | PKP2 |
| 23 | M | 46 | 1 | 0 | 0 | 0 | 1 | 0 | 1 | 0 | 0 | 0 | 0 | 0 | 0 | 1 | 2M, 1m | DSP |
| 24 | M | 36 | 0 | 1 | 0 | 0 | 0 | 0 | 0 | 1 | 1 | 0 | 0 | 1 | 0 | 0 | 2M, 2m | DSC2 |
| 25 | M | 49 | 1 | 0 | 1 | 0 | 0 | 0 | 0 | 0 | 1 | 0 | 0 | 1 | 0 | NA | 3M, 1m | DSC2 |
| 26 | M | 54 | 0 | 0 | 0 | 1 | 0 | 0 | 0 | 0 | 1 | 0 | 0 | 0 | 0 | 1 | 2M | DSG2 |
| 27 | F | 8 | 0 | 0 | 0 | 0 | 0 | 0 | 0 | 0 | 0 | 0 | 0 | 1 | 0 | 1 | 2M | DSP |
| 28 | M | 48 | 1 | 0 | 0 | 0 | 1 | 0 | 0 | 1 | 0 | 0 | 1 | 0 | 0 | NA | 1M, 3m | PKP2 |
| 29 | M | 35 | 1 | 0 | 0 | 1 | 0 | 0 | 0 | 0 | 0 | 1 | 0 | 0 | 0 | NA | 2M, 1m | DSP/PKP2 |
| 30 | M | 38 | 1 | 0 | 0 | 0 | 0 | 0 | 0 | 0 | 1 | 0 | 0 | 0 | 0 | 1 | 2M | PKP2 |
| 31 | M | 61 | 1 | 1 | 1 | 0 | 0 | 0 | 0 | 0 | 0 | 0 | 1 | 0 | 0 | NA | 1M, 2m | PKP2 |
| 32 | M | 15 | 1 | 0 | 0 | 0 | 0 | 1 | 0 | 0 | 0 | 0 | 1 | 0 | 0 | NA | 1M, 2m | PKP2 |
| 33 | M | 63 | 1 | 1 | 0 | 0 | 0 | 0 | 0 | 0 | 1 | 0 | 0 | 0 | 0 | 1 | 2M, 1m | PKP2 |
| 34 | M | 55 | 1 | 0 | 0 | 0 | 0 | 0 | 0 | 0 | 1 | 0 | 0 | 0 | 1 | NA | 2M, 1m | PKP2 |
| 35 | F | 39 | 1 | 0 | 0 | 1 | 0 | 0 | 0 | 1 | 0 | 0 | 1 | 0 | 0 | 0 | 2M, 2m | PKP2 |
| 36 | M | 60 | 1 | 0 | 0 | 0 | 1 | 0 | 0 | 1 | 1 | 0 | 0 | 0 | 0 | 1 | 2M, 2m | PKP2/ DSC2 |
| 37 | M | 12 | 1 | 1 | 0 | 1 | 0 | 0 | 0 | 0 | 0 | 0 | 0 | 0 | 0 | 1 | 2M, 1m | DSP |
| 38 | F | 24 | 1 | 0 | 0 | 0 | 1 | 0 | 0 | 0 | 0 | 1 | 0 | 0 | 0 | 0 | 1M 2m | DSC2 |
| 39 | M | 39 | 1 | 0 | 0 | 1 | 0 | 0 | 1 | 0 | 0 | 0 | 1 | 0 | 0 | 1 | 3M, 1m | PKP2 |
| 40 | F | 30 | 1 | 0 | 0 | 0 | 1 | 0 | 0 | 1 | 0 | 0 | 1 | 0 | 0 | 0 | 1M, 3m | PKP2 |
| 41 | M | 41 | 0 | 1 | 0 | 0 | 0 | 0 | 0 | 0 | 0 | 0 | 1 | 1 | 0 | 0 | 1M, 2m | DSG2 |
| 42 | F | 55 | 0 | 0 | 0 | 0 | 0 | 0 | 0 | 0 | 1 | 0 | 1 | 1 | 1 | NA | 2M, 2m | DSP |
| 43 | F | 13 | 0 | 0 | 0 | 1 | 0 | 0 | 0 | 0 | 1 | 0 | 1 | 1 | 0 | 0 | 3M, 1m | PKP2 |
| 44 | M | 13 | 1 | 0 | 0 | 1 | 0 | 0 | 0 | 1 | 0 | 0 | 1 | 0 | 0 | 1 | 2M, 2m | PKP2 |
| 45 | M | 64 | 0 | 1 | 0 | 1 | 0 | 0 | 0 | 0 | 0 | 0 | 1 | 0 | 0 | NA | 1M, 2m | PKP2 |
| 46 | M | 44 | 1 | 0 | 0 | 1 | 0 | 0 | 0 | 1 | 1 | 0 | 0 | 0 | 0 | 1 | 3M, 1m | PKP2 |
| 47 | M | 44 | 1 | 0 | 0 | 1 | 0 | 0 | 1 | 0 | 0 | 0 | 1 | 0 | 0 | 1 | 3M, 1m | None |
| 48 | F | 40 | 0 | 0 | 0 | 1 | 0 | 0 | 0 | 0 | 0 | 0 | 0 | 1 | 0 | 0 | 2M | None |
| 49 | M | 36 | 1 | 0 | 0 | 0 | 0 | 0 | 0 | 1 | 0 | 1 | 0 | 0 |  | 1 | 1M, 2m | None |
| 50 | M | 30 | 1 | 1 | 1 | 1 | 0 | 0 | 0 | 0 | 1 | 0 | 0 | 0 | 0 | NA | 3M, 1m | None |
| 51 | M | 30 | 1 | 0 | 0 | 1 | 0 | 0 | 0 | 0 | 0 | 0 | 1 | 0 | 0 | NA | 2M, 1m | None |
| 52 | M | 25 | 1 | 0 | 0 | 1 | 0 | 0 | 0 | 1 | 0 | 1 | 0 | 0 | 0 | 1 | 2M, 2m | None |
| 53 | M | 46 | 0 | 0 | 1 | 0 | 0 | 0 | 0 | 0 | 0 | 0 | 0 | 1 | 0 | NA | 2M | None |
| 54 | M | 13 | 1 | 0 | 0 | 1 | 0 | 0 | 0 | 0 | 0 | 0 | 0 | 0 | 0 | NA | 2M | None |
| 55 | M | 49 | 0 | 0 | 1 | 0 | 0 | 0 | 0 | 1 | 0 | 1 | 0 | 0 | 0 | NA | 1M, 2m | None |
| 56 | F | 42 | 1 | 1 | 0 | 0 | 0 | 0 | 0 | 0 | 0 | 0 | 0 | 0 | 0 | 1 | 1M 2m | None |
| 57 | F | 39 | 0 | 0 | 0 | 1 | 0 | 0 | 0 | 0 | 0 | 0 | 0 | 1 | 0 | 0 | 2M | None |
| 58 | M | 38 | 1 | 0 | 0 | 1 | 0 | 0 | 0 | 0 | 0 | 0 | 1 | 0 | 0 | 0 | 2M, 1m | None |
| 59 | F | 51 | 0 | 0 | 0 | 1 | 0 | 0 | 0 | 1 | 0 | 0 | 1 | 0 | 0 | NA | 1M, 2m | None |
| 60 | F | 41 | 0 | 0 | 0 | 1 | 0 | 0 | 0 | 0 | 0 | 0 | 1 | 0 | 1 | 0 | 1M, 2m | None |
| 61 | M | 46 | 1 | 0 | 0 | 0 | 0 | 0 | 0 | 0 | 1 | 0 | 0 | 0 | 0 | 0 | 2M | None |
| 62 | M | 61 | 1 | 0 | 0 | 0 | 0 | 0 | 0 | 0 | 1 | 0 | 0 | 0 | 0 | 0 | 2M | None |
| 63 | M | 48 | 0 | 0 | 0 | 1 | 0 | 0 | 0 | 0 | 1 | 0 | 0 | 0 | 0 | 0 | 2M | None |
| 64 | M | 22 | 1 | 0 | 0 | 1 | 0 | 0 | 0 | 0 | 0 | 0 | 1 | 0 | 0 | 1 | 2M, 1m | None |
| 65 | F | 21 | 1 | 0 | 0 | 1 | 0 | 0 | 0 | 1 | 0 | 0 | 0 | 0 | 0 | 1 | 2M, 1m | None |
| 66 | F | 23 | 1 | 0 | 1 | 1 | 0 | 0 | 1 | 0 | 1 | 0 | 0 | 0 | 0 | 1 | 5M | None |
| 67 | F | 47 | 0 | 0 | 1 | 0 | 0 | 0 | 0 | 1 | 0 | 1 | 0 | 0 | 0 | 1 | 1M, 2m | None |
| 68 | M | 47 | 0 | 0 | 1 | 0 | 0 | 0 | 0 | 1 | 0 | 0 | 1 | 0 | 0 | 1 | 1M, 2m | None |
| 69 | F | 41 | 1 | 0 | 1 | 1 | 0 | 0 | 0 | 0 | 0 | 0 | 0 | 0 | 0 | 1 | 3M | None |
| 70 | F | 47 | 0 | 0 | 0 | 0 | 0 | 0 | 0 | 0 | 1 | 0 | 1 | 1 | 1 | NA | 2M, 2m | None |

Clinical data according to TFC of AC patients from Padua cohort (discovery + validation cohorts). M – male; F – female; 1 – presence; 0 – absence; RV – right ventricle; LV – left ventricle, LBBB - left bundle branch block; LE-CMR – late gadolinium enhancement on cardiac magnetic resonance; NA - not available, M/m - major/minor diagnostic criterion; NSVT/SVT - not-sustained/sustained ventricular tachycardia; LAX - parasternal long-axis view; PSAX - parasternal short-axis view; RBBB - right bundle branch block; RVOT - RV outflow tract; SAECG - signal-averaged ECG.

Table S2. Discovery cohort DE miRNAs shared between tissue and blood arrays.

| **Discovery cohort** | **Tissue array (n = 9)** | **Blood array (n = 9)** | **Consistent directionality of Log_2_FC** |
| --- | --- | --- | --- |
| miRNA | Log_2_FC (mean ±SEM) | Log_2_FC (mean ±SEM) | Yes/No |
| miR-122-5p | -5.344±0.59 | 3.221±0.30 | No |
| miR-133a-3p | -1.093±0.27 | -1.421±0.82 | Yes |
| miR-133b | -1.073±0.11 | -1.945±0.65 | Yes |
| miR-142-3p | 1.92±0.6 | -1.512±0.40 | No |
| miR-144-3p | 1.153±0.09 | -1.255±0.42 | No |
| miR-149-3p | -1.216±0.41 | -1.045±0.39 | Yes |
| miR-182-5p | -1.104±0.24 | 1.087±0.35 | No |
| miR-183-5p | -1.136±0.24 | 1.177±0.14 | No |
| miR-208a-3p | 1.451±0.21 | 2.210±0.21 | Yes |
| miR-494-3p | -2.863±0.49 | -1.057±0.31 | Yes |

10 DE miRNAs found in common in tissue and blood profiles of the discovery cohort. Log_2_FC (mean ±SEM) values of the 10 miRNAs assessed by 84 cardiac-related array. Consistent directionality of the DE in tissue versus blood.

Table S3. Thirteen miRNAs expression in the validation cohort.

| Validation cohort | | qPCR validation | | |
| --- | --- | --- | --- | --- |
| N | miRNA | AC *gen+* (n = 46)  Log_2_FC (mean ±SEM) | Ctrl (n = 20)  Log_2_FC (mean ±SEM) | p value  (Mann-Whitney test) |
| 1 | miR-122-5p | 1.870±0.20 | -0.110±0.15 | < 0.0001 |
| 2 | miR-133a-3p | -1.150±0.27 | 0.000±0.14 | < 0.0001 |
| 3 | miR-133b | -1.290±0.22 | -0.197±0.13 | < 0.0001 |
| 4 | miR-142-3p | -2.40±0.0.35 | 0.000±0.07 | < 0.0001 |
| 5 | miR-144-3p | 0.221±0.15 | 0.193±0.16 | 0.687 |
| 6 | miR-149-3p | -0.021±0.14 | 0.201±0.11 | 0.113 |
| 7 | miR-182-5p | 0.780±0.19 | 0.000±0.20 | 0.004 |
| 8 | miR-183-5p | 0.710±0.12 | 0.000±0.19 | 0.025 |
| 9 | miR-208a-3p | 0.249±0.14 | 0.275±0.22 | 0.052 |
| 10 | miR-494-3p | 0.195±0.16 | 0.003±0.28 | 0.817 |
| 11 | miR-21-5p | -0.213±0.12 | 0.051±0.12 | 0.136 |
| 12 | miR-320a | -0.221±0.12 | 0.109±0.14 | 0.067 |
| 13 | miR-184 | -0.337±0.18 | 0.115±0.10 | 0.072 |

Log_2_FC (mean ±SEM) values of the 13 miRNA analysed on 46 AC gen+ and 20 controls (validation cohort); p value calculated by Mann-Whitney test.

**Supplemental Figure Legends**

**Figure S1**: DE miRNAs in the AC-tissue profile and blood profile on the discovery cohort by the 84 cardiac-related array (Log2FC, mean ±SEM).

**Figure S2**: Gene-dependent miRNA analysis. A: Clustergram of the 84 cardiac-related array analysis on the 9 samples divided based on mutated gene; group 1 – PKP2, group 2 – DSP, group 3 – DSG2. B: DE miRNAs of PKP2 profile. C: DE miRNAs of DSP profile. D: DE miRNAs of DSG2 profile; Log2FC, mean ±SEM.

**Supplementary bibliography**

1. Vlachos, I. S.; Zagganas, K.; Paraskevopoulou, M. D.; Georgakilas, G.; Karagkouni, D.; Vergoulis, T.; Dalamagas, T.; Hatzigeorgiou, A. G., DIANA-miRPath v3.0: deciphering microRNA function with experimental support. *Nucleic Acids Res* **2015,** *43* (W1), W460-6.

2. Vlachos, I. S.; Paraskevopoulou, M. D.; Karagkouni, D.; Georgakilas, G.; Vergoulis, T.; Kanellos, I.; Anastasopoulos, I. L.; Maniou, S.; Karathanou, K.; Kalfakakou, D.; Fevgas, A.; Dalamagas, T.; Hatzigeorgiou, A. G., DIANA-TarBase v7.0: indexing more than half a million experimentally supported miRNA:mRNA interactions. *Nucleic Acids Res* **2015,** *43* (Database issue), D153-9.

3. Paraskevopoulou, M. D.; Georgakilas, G.; Kostoulas, N.; Vlachos, I. S.; Vergoulis, T.; Reczko, M.; Filippidis, C.; Dalamagas, T.; Hatzigeorgiou, A. G., DIANA-microT web server v5.0: service integration into miRNA functional analysis workflows. *Nucleic Acids Res* **2013,** *41* (Web Server issue), W169-73.

4. Chou, C. H.; Shrestha, S.; Yang, C. D.; Chang, N. W.; Lin, Y. L.; Liao, K. W.; Huang, W. C.; Sun, T. H.; Tu, S. J.; Lee, W. H.; Chiew, M. Y.; Tai, C. S.; Wei, T. Y.; Tsai, T. R.; Huang, H. T.; Wang, C. Y.; Wu, H. Y.; Ho, S. Y.; Chen, P. R.; Chuang, C. H.; Hsieh, P. J.; Wu, Y. S.; Chen, W. L.; Li, M. J.; Wu, Y. C.; Huang, X. Y.; Ng, F. L.; Buddhakosai, W.; Huang, P. C.; Lan, K. C.; Huang, C. Y.; Weng, S. L.; Cheng, Y. N.; Liang, C.; Hsu, W. L.; Huang, H. D., miRTarBase update 2018: a resource for experimentally validated microRNA-target interactions. *Nucleic Acids Res* **2018,** *46* (D1), D296-D302.

5. Xia, J.; Gill, E. E.; Hancock, R. E., NetworkAnalyst for statistical, visual and network-based meta-analysis of gene expression data. *Nat Protoc* **2015,** *10* (6), 823-44.
