# Supplementary material for: A microRNA Expression Profile as Non-Invasive Biomarker in a Large Arrhythmogenic Cardiomyopathy Cohort
Source: Int J Mol Sci. 2020 Feb 24;21(4):1536. doi: 10.3390/ijms21041536 (PMC7073183; doi:10.3390/ijms21041536)
Supplement: Supplementary file 1 [file ijms-21-01536-s001.zip › suppFigures_IJMS.pptx]

## Slide 1
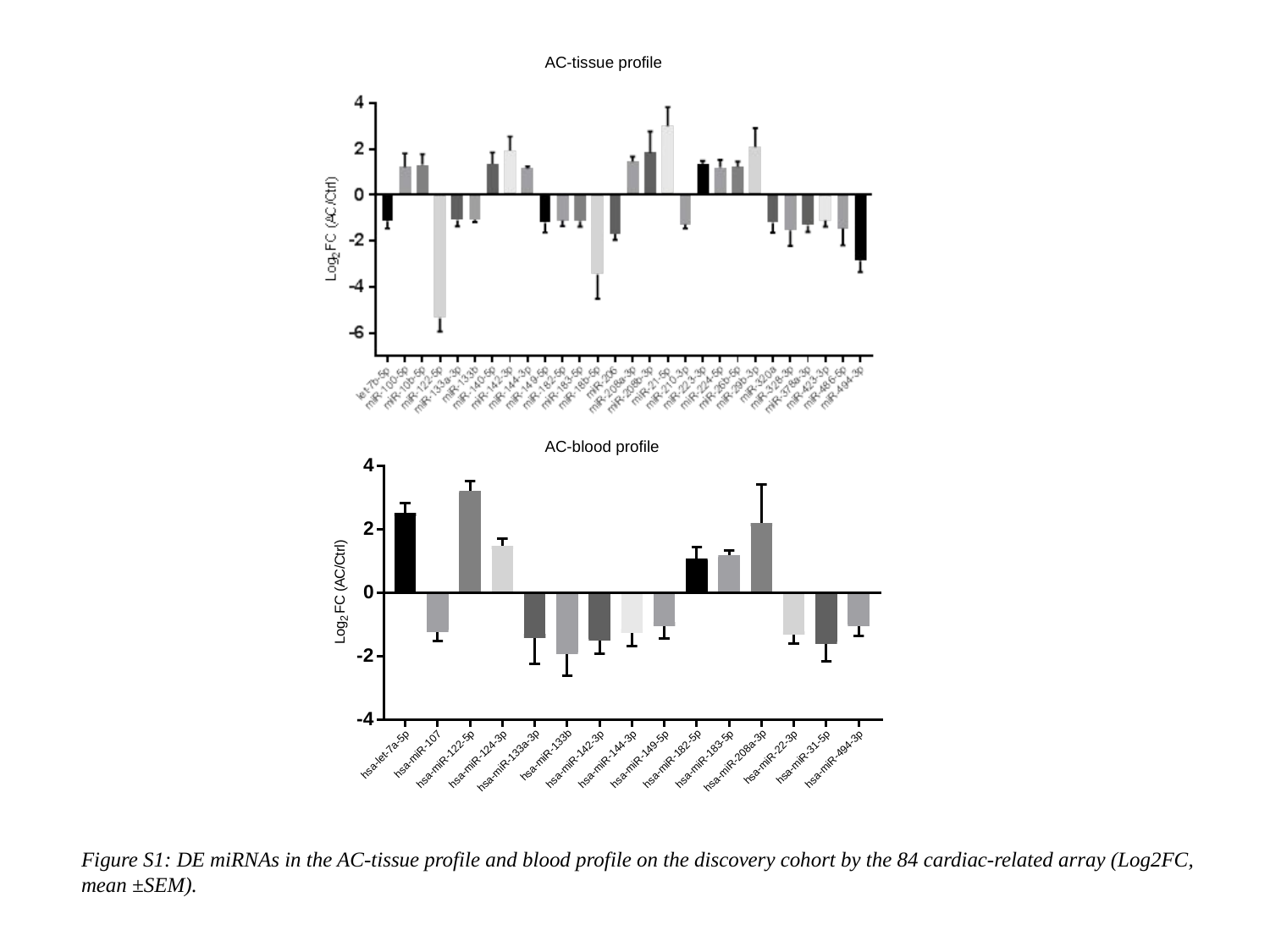

AC-tissue profile
AC-blood profile
Figure S1: DE miRNAs in the AC-tissue profile and blood profile on the discovery cohort by the 84 cardiac-related array (Log2FC, mean ±SEM).

## Slide 2
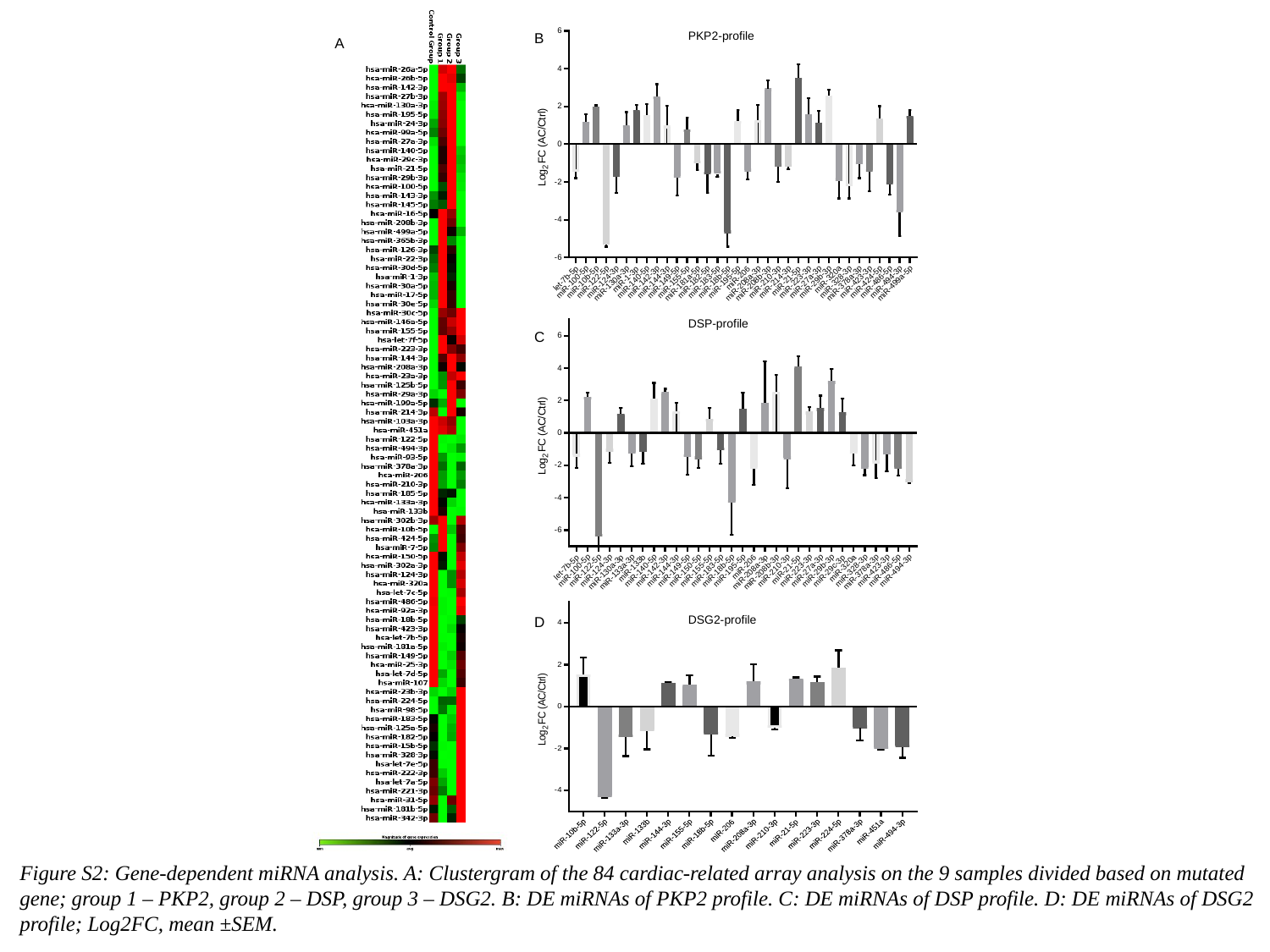

B
PKP2-profile
A
DSP-profile
C
DSG2-profile
D
Figure S2: Gene-dependent miRNA analysis. A: Clustergram of the 84 cardiac-related array analysis on the 9 samples divided based on mutated gene; group 1 – PKP2, group 2 – DSP, group 3 – DSG2. B: DE miRNAs of PKP2 profile. C: DE miRNAs of DSP profile. D: DE miRNAs of DSG2 profile; Log2FC, mean ±SEM.
